# Medical Missionaries

**Published:** 1901-07-20

**Authors:** 


### MEDICAL MISSIONARIES.

DR. WILLIAM ELDER, of Bristol, writes:—

I have been a constant reader of THE HOSPITAL for some time now, and having been a medical missionary for 30 years, first in Edinburgh, then in India, and now in Bristol, I feel I must venture on a little remonstrance anent your article of July 6 headed "The Medical Missionary!" You seem to me to be a little hard on the principle of medical missions, which have for their keynote—"Heal the sick and say unto them the Kingdom of God is coming nigh unto you," or, in other words, do your very best for the body and soul of your patient. The medical missionary does not look on his patient simply as "a case" out of which he expects to make so much money, but as a human being, requiring something more than the diagnosis of his disease and the application of remedies. I am not one of those, however, who write and speak at times as if the medical work of a medical missionary were simply a peg on which to hang an opportunity of preaching the Gospel! In my mind, unless there be a medical necessity, medical missions are a misnomer and savour of Jesuitism; but given a medical necessity then I hold that there is no form of Christian service to one's fellows which comes nearer to the spirit of Him who went about continually doing good, and who, as far as I can understand, healed because folks were ill and not simply to make known His divinity. To my mind in every act of healing He demonstrated the depth of sympathy He had with suffering humanity.

Then you hint that the medical missionary in heathen lands is bound to be a proselytiser, if he be an honest missionary! But the same objection could be taken to the doctor pure and simple, and also to the educationalist, in India at least, for Western education and Western medicine are quite opposed to the religion of the Hindoo; and I suppose the same might be said of the Chinaman.

[We put the matter as sympathetically as was possible, but we are sure that it is not absolutely clear that the position of intimacy and trust existing between doctor and patient can be properly used by the doctor for his own purposes, however well meaning such purposes may be.—ED. THE HOSPITAL.]

